# Robust free-breathing SASHA T_1_ mapping with high-contrast image registration

**DOI:** 10.1186/s12968-016-0267-9

**Published:** 2016-08-17

**Authors:** Kelvin Chow, Yang Yang, Peter Shaw, Christopher M. Kramer, Michael Salerno

**Affiliations:** 1 Department of Medicine, University of Virginia Health System, PO Box 800158, 1215 Lee St, Charlottesville, 22908 VA USA; 2 Department of Biomedical Engineering, University of Virginia Health System, Charlottesville, VA USA; 3 Department of Radiology and Medical Imaging, University of Virginia Health System, Charlottesville, VA USA

**Keywords:** T_1_ mapping, SASHA, Free-breathing, Image registration, Extracellular volume, Fibrosis

## Abstract

**Background:**

Many widely used myocardial T_1_ mapping sequences use breath-hold acquisitions that limit the precision of calculated T_1_ maps. The SAturation-recovery single-SHot Acquisition (SASHA) sequence has high accuracy with robustness against systematic confounders, but has poorer precision compared to the commonly used MOdified Look-Locker Inversion recovery (MOLLI) sequence. We propose a novel method for generating high-contrast SASHA images to enable a robust image registration approach to free-breathing T_1_ mapping with high accuracy and precision.

**Methods:**

High-contrast (HC) images were acquired in addition to primary variable flip angle (VFA) SASHA images by collecting an additional 15 k-space lines and sharing k-space data with the primary image. The number of free-breathing images and their saturation recovery times were optimized through numerical simulations. Accuracy and precision of T_1_ maps using the proposed SASHA-HC sequence was compared in 10 volunteers at 1.5 T to MOLLI, a breath-hold SASHA-VFA sequence, and free-breathing SASHA-VFA data processed using conventional navigator gating and standard image registration. Free-breathing T_1_ maps from 15 patients and 10 volunteers were graded by blinded observers for sharpness and artifacts.

**Results:**

Difference images calculated by subtracting HC and primary SASHA images had greater tissue-blood contrast than the primary images alone, with a 3× improvement for 700 ms TS saturation recovery images and a 6× increase in tissue-blood contrast for non-saturated images. Myocardial T_1_s calculated in volunteers with free-breathing SASHA-HC were similar to standard breath-hold SASHA-VFA (1156.1 ± 28.1 ms vs 1149.4 ± 26.5 ms, *p* >0.05). The standard deviation of myocardial T_1_ values using a 108 s free-breathing SASHA-HC (36.2 ± 3.1 ms) was 50 % lower (*p* <0.01) than breath-hold SASHA-VFA (72.7 ± 8.0 ms) and 34 % lower (*p* <0.01) than breath-hold MOLLI (54.7 ± 5.9 ms). T_1_ map quality scores in volunteers were higher with SASHA-HC (4.7 ± 0.3 out of 5) than navigator gating (3.6 ± 0.4, *p* <0.01) or normal registration (3.7 ± 0.4, *p* <0.01). SASHA-HC T_1_ maps had comparable precision to breath-hold MOLLI using a retrospectively down-sampled 30 s free-breathing acquisition and 30 % higher precision with a 60 s acquisition.

**Conclusions:**

High-contrast SASHA images enable a robust image registration approach to free-breathing T_1_ mapping. Free-breathing SASHA-HC provides accurate T_1_ maps with higher precision than MOLLI in acquisitions longer than 30 s.

**Electronic supplementary material:**

The online version of this article (doi:10.1186/s12968-016-0267-9) contains supplementary material, which is available to authorized users.

## Background

Reliable assessment of increased myocardial extracellular volume, often due to diffuse interstitial fibrosis, is of significant clinical interest due to its ubiquitous presence in many cardiac diseases. Recent studies have shown altered native myocardial T_1_ values in patients with a wide range of diseases including patients with ST-segment elevation myocardial infarction (STEMI) and non-STEMI [1] and hypertrophic and dilated cardiomyopathy [2], as well as infiltrative diseases such as amyloidosis [3, 4], iron overload [5], and Anderson-Fabry disease [6–8]. T_1_ mapping with gadolinium contrast can also be used to estimate the extracellular volume fraction (ECV), a physiologically relevant parameter that increases with diffuse interstitial fibrosis and infiltrative diseases, and abnormal ECV values have been reported across a wide spectrum of cardiac diseases [9–11]. Correlations of abnormal T_1_ values with other biomarkers of myocardial remodeling have also been reported in asymptomatic patients with diabetes [12], aortic stenosis [13], and hypertension with left ventricular hypertrophy [14], suggesting T_1_ may also be a sensitive marker of early pre-clinical remodeling.

Reliable ECV quantification requires techniques with both high accuracy and precision. Accurate T_1_ mapping techniques without systematic confounders are intuitively desirable because they may be more specific to T_1_ changes associated with changes in ECV. While the MOdified Look-Locker Inversion recovery (MOLLI) technique [15, 16] has gained widespread adoption, it is sensitive to factors such as T_2_ [17], magnetization transfer [18], and off-resonance [19], and changes in any of these confounders result in changes in measured T_1_ values [20]. Saturation-recovery based sequence such SAturation-recovery single-SHot Acquisition (SASHA) [21], Saturation Method using Adaptive Recovery Times (SMART_1_Map) [22], and SAturation Pulse Prepared Heart rate independent Inversion-REcovery sequence (SAPPHIRE) [23] are more robust to these confounders, but their adoption has been limited by poorer precision which results from reduced dynamic range and signal-to-noise compared to the inversion-recovery based MOLLI sequence [20, 24]. Higher precision techniques with less variability are needed to reliably detect subtle T_1_ changes in individual patients and to better identify focal T_1_ abnormalities.

Free-breathing T_1_ mapping techniques can potentially increase precision compared to breath-hold techniques by acquiring more images over a longer duration, thus reducing uncertainty in calculated T_1_ values. Free-breathing approaches also extend the utility of T_1_ mapping to patients who are unable to adequately hold their breath, such as those with shortness of breath or heart failure. One common approach for addressing respiratory motion during free-breathing T_1_ acquisitions is to use respiratory navigator triggering, such as in the high-resolution Accelerated and Navigator-Gated Look-Locker Imaging for cardiac T1 Estimation (ANGIE) T_1_ mapping sequence [25]. The position of the diaphragm is monitored using a separate acquisition and imaging is performed within a small window of respiratory phase. However, cardiac imaging with respiratory navigation may still have considerable residual motion because the heart and lung do not always move in perfect unison. Additionally, respiratory navigators can be challenging in routine clinical practice, as clinical patients often have irregular respiratory patterns that reduce navigator gating efficiency. An alternative approach to free-breathing imaging is to continuously acquire images in all respiratory phases and use image registration to align a subset of images. This approach has been successfully applied to late gadolinium enhancement [26] and T_2_^*^ imaging [27], but direct image registration of SASHA’s saturation recovery images is challenging due to poor tissue-blood contrast.

We propose to enable free-breathing SASHA T_1_ mapping using image registration by acquiring additional secondary images with higher tissue-blood contrast to improve registration performance. A modified image readout was designed to enhance tissue-blood contrast without affecting T_1_ accuracy, and the sampling scheme of saturation recovery times was optimized with simulations. Accuracy of the proposed high-contrast (HC) SASHA sequence was verified using phantom experiments. Free-breathing SASHA-HC was evaluated in a group of healthy volunteers and clinical patients with comparisons to standard breath-hold SASHA and MOLLI in volunteers.

### Theory

**Fig. 1:**
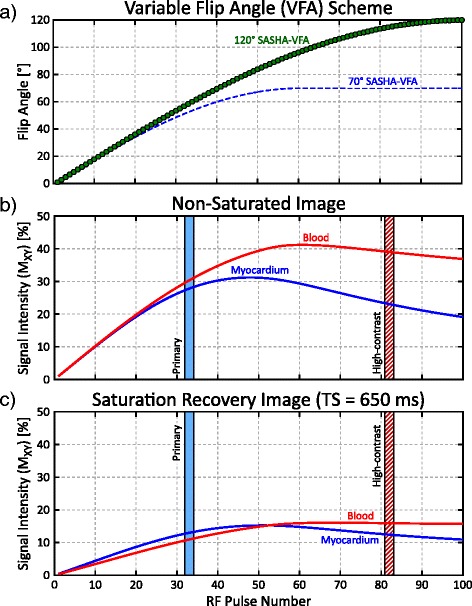
Bloch equation simulation of a sinusoidal-ramped variable flip angle (VFA) bSSFP readout for native myocardium (T_1_/T2 = 1175/50 ms) and blood (T_1_/T2 = 1650/240 ms). **a** Readout flip angles are increased in a sinusoidal ramp pattern for 120° SASHA-VFA (*green circles*) and 70° SASHA-VFA (*dashed blue line*). **b** Signal evolution of myocardium (*blue*) and blood (*red*) for a non-saturated image using 120° SASHA-VFA. Typical k-space center locations for the primary and high-contrast images are marked with light blue and dashed red boxes respectively. **c** Signal evolution for a saturation recovery image using 120° SASHA-VFA

The SASHA sequence consists of a set of single-shot balanced steady-state free precession (bSSFP) images with various saturation recovery preparation times (TS) or without saturation preparation (non-saturated, NS) [21]. Variable flip angle (VFA) ramping of the flip angles in the bSSFP readout is used to reduce the severity of image artifacts and increase T_1_ precision by enabling 2-parameter model calculation of T_1_ values with minimal systematic errors [28]. A Bloch equation simulation of transverse magnetization for native blood and myocardium during a VFA readout (Fig. 1) shows the time evolution of tissue-blood contrast. Conventional SASHA-VFA images have poor tissue-blood contrast because the k-space center is acquired early in the readout, prior to significant tissue-blood signal intensity differences, in order to minimize bias in derived T_1_ values. Images acquired with a later center k-space would have better contrast, but T_1_ maps calculated using this data would have larger systematic errors.

**Fig. 2:**
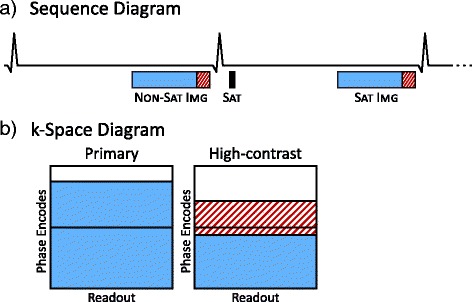
**a** Partial sequence diagram of the proposed high-contrast SASHA sequence showing a non-saturated image and a saturation recovery image. Image acquisition is extended by ~40 ms by appending additional k-space lines (*dashed red*) to primary image readouts (*light blue*). **b** k-space coverage is shown for primary and high-contrast images. The lower half of k-space is shared between primary and high-contrast images and the additional k-space lines cover the central portion of k-space

We propose that high-contrast (HC) images can be acquired immediately following the standard primary image acquisition. A schematic diagram of the proposed sequence and k-space coverage is shown in Fig. 2. The HC image acquisition consists of a small number of additional k-space lines at a higher acceleration rate covering the central portion of k-space. This data can be keyhole shared with high-frequency k-space data from the primary image (Fig. 2), as they are acquired immediately prior and have similar contrast. The temporal similarity and shared data between the images ensures that they are intrinsically co-registered, thus allowing image registration performed on HC images to be directly applied to the primary images.

## Methods

### Sequence design

The VFA modulation pattern follows a sinusoidal increase in flip angles in order to minimize off-resonance artifacts (Fig. 1). Lower initial flip angles minimize T_2_-dependent errors in SASHA T_1_ values when a 2-parameter model is used. In order to maximize the contrast of the HC image, a higher 120° final flip angle was used with a longer duration of VFA modulation, optimized to have similar accuracy characteristics as the original VFA pattern with a 70° final flip angle [28]. Bloch equation simulations incorporating slice profile effects were used to characterize the tissue-blood contrast of the proposed VFA scheme. Simulations used a T_1_/T_2_ of 1175/50 ms for native myocardium and a T_1_/T_2_ of 1650/240 ms for native blood [21].

General sequence parameters included: 1.19/2.76 ms TE/TR, 120° maximum flip angle, 0.6 ms Hanning-weighted sinc excitation RF pulses, 360 × 270 mm field of view, 256 × 150 matrix size, 7/8^ths^ partial Fourier, 78 % phase resolution, and rate 2 acceleration for a total of 65 phase encodes for the primary image. The high-contrast acquisition consisted of 15 additional phase encodes at rate 3 acceleration, asymmetrically distributed across center k-space with 11 lines in the top half of k-space (Fig. 2). Thirty-six fully-sampled k-space lines were acquired in a separate heartbeat for parallel imaging reconstruction calibration.

### Sampling scheme optimization

Precision, i.e. uncertainty, in T_1_ maps is determined by the total number of images used in its calculation as well as the sampling of the recovery curve, as defined by the TS times [29, 30]. For a 2-parameter model of T_1_ fitting that assumes ideal saturation, the optimal pattern for a breath-hold acquisition is a single NS image followed by all remaining images at a fixed TS time less than the R-R interval [30]. Images with longer TS times that required multiple heartbeats were not found to improve precision for breath-hold imaging due to a corresponding reduction in the total number of acquired images.

Longer imaging times in free-breathing acquisitions allow more flexibility in sampling strategies, but any given image may be discarded if it is at a different respiratory phase than the rest of the images. To maximize sampling redundancy, a scheme consisting of only NS images and TS images less than an R-R interval was chosen. The optimal TS time and ratio of NS to TS images was determined by extending the analytical framework developed by Kellman et al. [30]. Simulations assumed TS images were acquired in every heartbeat and NS images required 4 additional non-imaging heartbeats for complete magnetization recovery, thus requiring 5 heartbeats each. The first NS image did not require non-imaging heartbeats, as it was assumed to be the first acquired image. For example, a 45 heartbeat acquisition duration would allow for 1 NS image and 44 TS images, 2 NS and 39 TS images, 3 NS and 34 TS images, and so forth. Simulations used a heart rate of 60 bpm and higher heart rates would increase the number of images that could be acquired per unit time, thus increasing acquisition efficiency and reducing uncertainty in the calculated T_1_ values.

### Image reconstruction

GRAPPA reconstruction [31] was used to fill in the undersampled k-space data for primary (solid blue, Fig. 2) and high-contrast (dashed red lines, Fig. 2) acquisitions. High-contrast k-space data was then combined with the lower part of k-space from the primary data and projection over convex sets (POCS) [32] was used to fill in the missing upper part of k-space. Difference images were calculated by subtracting the primary and HC images to further improve tissue-blood contrast.

### Phantom studies

Accuracy of the proposed SASHA-HC sequence was compared against gold standard spin-echo T_1_ measurements in a set of NiCl_2_-doped agar phantoms (T_1_MES) [33]. Inversion-recovery spin-echo measurements were performed on a 1.5 T Siemens MAGNETOM Avanto (Siemens Healthcare, Erlangen, Germany) scanner with a 300 × 131 mm field of view, 192 × 84 matrix size, 10 mm slice thickness, 90° flip angle, 11 ms echo time (TE), 19 inversion times (TI) spanning 50–3000 ms, and 10 s repetition time with one acquired k-space line per repetition. T_2_ values were characterized using a spin-echo sequence with 8 TEs spanning 11–250 ms and other parameters as above. SASHA-VFA was acquired with both a 70° maximum flip angle and a 120° maximum flip angle as used in SASHA-HC. Both SASHA sequences had sequence parameters as described above with a NS image and 10 images with a TS of 600 ms. All SASHA sequences used an optimized 6-pulse train saturation design with <1 % residual magnetization over the range of off-resonance (B_0_) and radiofrequency (B_1_) scale factors expected at 1.5 T and 3 T [34].

### In-vivo studies

Ten healthy volunteers were imaged at 1.5 T with written informed consent and approval from the University of Virginia Institutional Review Board for Health Sciences Research. T_1_ mapping was performed on a single mid-ventricular short-axis slice using free-breathing 120° SASHA-VFA, breath-hold 120° SASHA-VFA, standard breath-hold 70° SASHA-VFA, and MOLLI sequences. Free-breathing 120° SASHA-VFA had 10 non-saturated images and 50 images with the longest TS time allowed by the subject’s heart rate with other sequence parameters as described above. Respiratory position for each image was monitored using a conventional crossed-beam 1D navigator placed at the dome of the right hemi-diaphragm. Navigators were acquired immediately prior to the primary image for all SASHA acquisitions and were oriented to avoid overlap with the heart. An acceptance window of ±4 mm was prospectively prescribed, but navigator data was not used to trigger or re-acquire images.

Breath-held sequences were acquired during end-expiratory breath-holds with 1 non-saturated image and 10 TS images for SASHA-VFA with parameters as described above. MOLLI T_1_ mapping was performed using a 5(4)3 acquisition scheme with a 121 ms minimum TI, 80 ms TI increment, 1.12/2.68 ms TE/TR, 35° flip angle, and matched resolution to SASHA-VFA.

T_1_ mapping with free-breathing 120° SASHA-VFA was performed on 15 patients referred for clinical CMR examination or enrolled in ongoing clinical studies of ischemic heart disease or heart failure. T_1_ mapping was also performed following contrast injection when permitted by the clinical protocol, using a fixed TS time that was decided based upon amount of contrast agent given and time elapsed following injection.

### In-vivo image analysis

SASHA T_1_ maps were calculated using a 2-parameter model assuming ideal saturation and MOLLI T_1_ maps were calculated using a 3-parameter model with Look-Locker correction [15, 35]. Free-breathing SASHA-VFA data was analyzed by using conventional respiratory navigator data (NAV) to select end-expiratory images using a ±4 mm acceptance window. Image registration was not used for breath-hold or navigator-gated data.

**Fig. 3:**
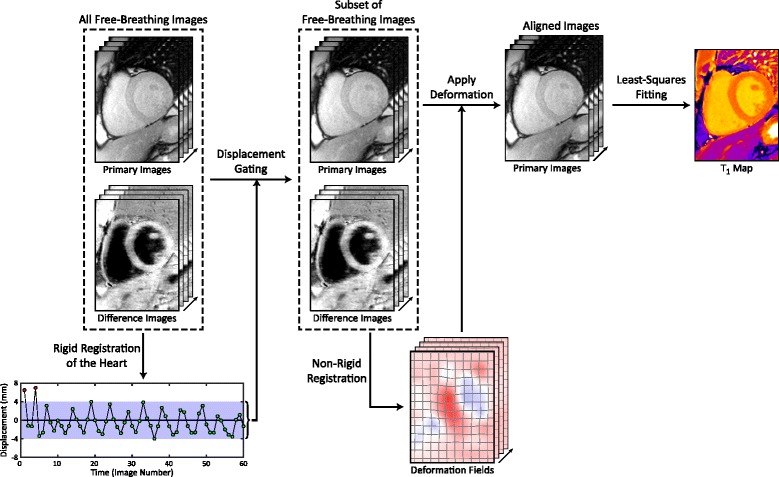
Overview of proposed analysis for free-breathing SASHA-HC. The displacement of the heart in free-breathing data is estimated using rigid image registration and used to select a subset of images for further analysis. This subset is aligned with a non-rigid image registration algorithm using both primary and difference images, and the resulting deformation fields are applied to the primary images. A T_1_ map is calculated from the aligned images using non-linear least squares curve fitting

The relative respiratory phase of free-breathing SASHA images was also estimated using the average displacement of the heart in the principal direction of motion, as defined by the direction with the highest singular value following singular value decomposition of the displacement matrix. Pair-wise affine registration was applied to a small image region around the heart and displacement was averaged over the entire region. An acceptance window of ±4 mm was used to discard images at very different respiratory phases due to the potential for through-plane motion and mis-registration artifacts. The position of the acceptance window was determined automatically to maximize the number of selected images. The subset of remaining images was aligned using the Advanced Normalization Tools (ANTs) software with a non-rigid image registration algorithm [36], using information from both the difference and primary images simultaneously (HC-REG). Images were also aligned using information from only the primary images (NORM-REG) to determine the additive value of high-contrast difference images. Aligned images were obtained by applying the calculated deformation field using cubic spline interpolation in a single step to reduce blurring from repeated interpolation. The overall proposed analysis for free-breathing SASHA with displacement gating and HC registration is summarized in Fig. 3.

The left ventricular (LV) myocardium from each T_1_ map was manually segmented by identifying the endocardial and epicardial borders and a blood pool contour was drawn in the LV cavity. Contouring was performed using custom analysis software that allowed visualization of contours on both T_1_ maps and NS/TS images simultaneously. Myocardial contours were conservatively drawn with a narrow window level on the T_1_ colormap to minimize partial voluming effects and regions with artifacts on the NS/TS images were avoided. Myocardial and blood T_1_ values were characterized by the mean and standard deviation of pixel map T_1_ values within their respective contours. Tissue-blood contrast was characterized as the difference between myocardial and blood signal intensities. The change in contrast for both primary SASHA images and difference images was characterized by the ratio of tissue-blood differences for NS and TS images respectively.

Free-breathing SASHA data aligned using HC-REG were further analyzed to determine the in-vivo relationship between the precision of calculated T_1_ maps and the number of raw images used. Additional T_1_ maps were calculated from each dataset using incrementally fewer NS and TS images and the images selected were randomly chosen to reduce the influence of artifacts or mis-registration of any given image. A boot-strapping approach was used where this selection process was repeated 10 times for each combination of NS and TS images and the standard deviation of myocardial T_1_ values was averaged over the 10 repetitions.

### Image quality evaluation

Free-breathing SASHA T_1_ maps from the 10 healthy volunteers and 15 clinical subjects reconstructed using NAV, NORM-REG, and HC-REG were evaluated by two experienced cardiologists on a 5-point scale (1-poor to 5-excellent). Cardiologists were blinded to the reconstruction method and asked to focus both on the presence of artifacts and the sharpness of the myocardial borders while grading.

### Statistical methods

Statistical analysis was performed in SAS 9.4 (SAS Institute, Cary, North Carolina, USA). Continuous variables and image quality scores are reported as mean ± standard deviation. Comparisons between the blood and myocardial T_1_ values derived from different T_1_ mapping techniques were performed using linear mixed models (PROC MIXED) with subjects treated as a random factor. A similar analysis was performed for the image quality scores, controlling for the reader as a fixed effect. Normality assumptions were verified using q-q plots to visually assess for significant departures from normality. Statistical significance was set at *p* <0.05 with Tukey-Kramer adjustments for multiple comparisons where applicable.

## Results

### Simulations

**Fig. 4:**
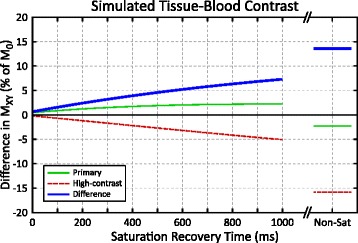
Bloch equation simulation of tissue-blood contrast for primary (*thin green*), high-contrast (*dashed red*), and difference (*thick blue*) images as a function of saturation recovery time. Contrast is characterized by the difference between myocardial and blood transverse magnetization (M_XY_)

Bloch equation simulations show limited tissue-blood contrast for primary images, with reversed contrast between saturation recovery and non-saturated images (green line, Fig. 4). High-contrast images have greater contrast and blood is brighter than the myocardium for both NS and TS images. Subtracting the primary and high-contrast images to produce a difference image further improves contrast for the saturation recovery images, with a 3× increase in contrast compared to the primary images for a 700 ms TS time. Tissue-blood contrast increases for all images with longer TS times and non-saturated images had a 6× increase in contrast.

**Fig. 5:**
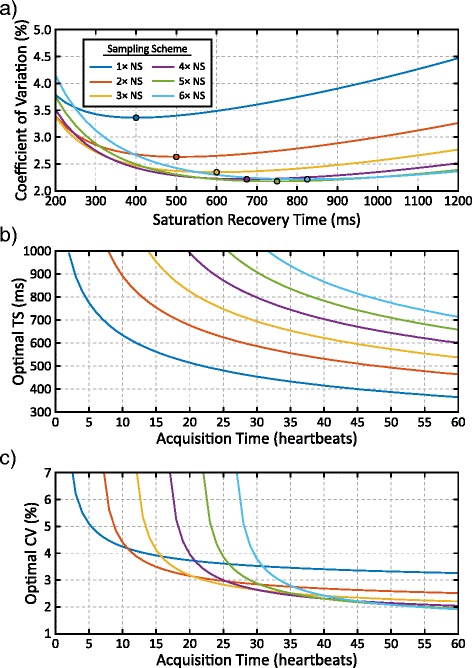
**a** Simulated precision for native myocardium for an acquisition duration of 45 heartbeats. The optimal TS times, corresponding to the minimum coefficient of variation, are marked for sampling scheme with various NS images. **b** The optimal TS times for each sampling schemes are plotted as a function of total acquisition duration. **c** The optimal coefficients of variation (CV) is plotted as a function of acquisition time, using fixed TS times from b). All simulations assume a 100 % acceptance rate and a heart rate of 60 bpm

Relationships between TS time, precision, and acquisition time are summarized in Fig. 5. For a fixed acquisition time of 45 s, the optimal TS (with the lowest coefficient of variation) increases with the number of NS images acquired (Fig. 5a). However, the loss in precision for sub-optimal TS times is relatively small when more NS images are utilized in the reconstruction, as shown by the flat response between the coefficient of variation and TS times between 600 and 1000 ms. The optimal TS time also decreases with increasing acquisition times (Fig. 5b). Schemes with more than 1 NS image have lower variability with acquisition times longer than 11 heartbeats and schemes with ≥2 NS images have similar performance for acquisition times >40 heartbeats (Fig. 5c).

### Phantom studies

The phantom contained 9 vials with T_1_ values spanning 259–1462 ms and T_2_ values spanning 45–251 ms, as determined by spin-echo measurements. The mean error in standard 70° SASHA-VFA was −0.7 ± 0.9 % and the mean error in 120° SASHA-VFA was 0.2 ± 1.1 %.

### In-vivo studies

Healthy subjects were aged 33 ± 9 yo (4 female, 6 male) with heart rates of 60 ± 7 bpm and patients were aged 61 ± 11 yo (11 female, 4 male) with heart rates of 64 ± 12 bpm. Free-breathing SASHA-HC acquisitions averaged 108 ± 7 s in healthy volunteers. The TS time used in native imaging was 644 ± 92 ms in volunteers and 616 ± 118 ms in patients. Post-contrast imaging was available in 12 of 15 clinical subjects with TS times of 342 ± 60 ms. The achievable maximum flip angle across all subjects was 119 ± 3°.

**Fig. 6:**
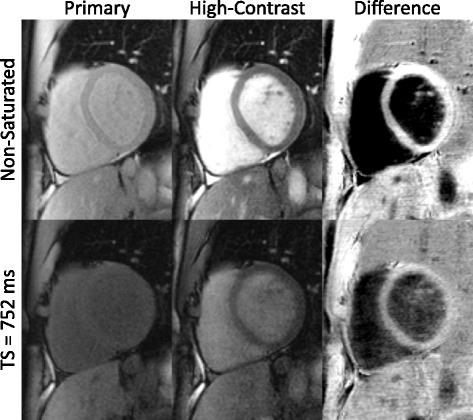
Primary, high-contrast, and difference images are shown for non-saturated and saturation recovery preparation in a healthy volunteer. Brightness and contrast levels in displayed images are matched between the NS and TS images

Primary, high-contrast, and difference images are shown for a healthy subject without gadolinium contrast in Fig. 6. The primary saturation recovery image has poorer tissue-blood contrast than the NS image and the contrast is reversed. High-contrast images improve contrast for both images with moderate contrast in the TS image. Difference images have the highest tissue-blood contrast and the contrast is consistent between NS and TS images. The average increase in tissue-blood contrast in the healthy volunteers was 5.7 ± 1.7× for NS and 6.1 ± 1.1× for TS images.

Navigator gating of free-breathing data in volunteers resulted in an acceptance rate of 49 ± 17 %, while the displacement gating had an acceptance rate of 91 ± 11 %. Mild to moderate residual motion was observed in most navigator-gated image series. NORM-REG had reasonable performance in correcting bulk motion of the heart, although misalignment of the septum was sometimes observed. Good quality image registration using HC-REG was observed for all subjects. Gating and image registration performance is visualized for one subject in Additional file 1: Video S1.

Free-breathing 120° SASHA-VFA T_1_ maps reconstructed with HC-REG (i.e. SASHA-HC) had higher image scores (4.7 ± 0.3 out of 5) than both NAV (3.6 ± 0.4, *p* <0.01) or NORM-REG (3.7 ± 0.4, *p* <0.01). Overall image scores were lower in patients (*p* <0.05), but similar trends were evident with HC-REG (4.1 ± 0.7) having higher scores than both NAV (3.5 ± 0.6) and NORM-REG (3.5 ± 0.8).

**Fig. 7:**
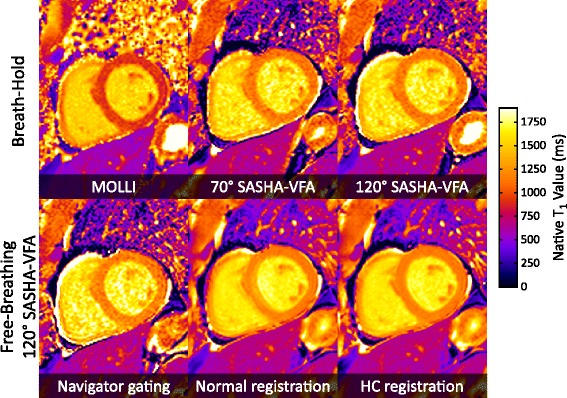
T_1_ maps from a healthy subject using different techniques. Breath-hold techniques are shown in the top row and the bottom row shows T_1_ maps calculated from free-breathing 120° SASHA-VFA data using various approaches

In-vivo T_1_ measurements from all sequences are summarized in Table 1 and T_1_ maps from one subject are shown in Fig. 7. Myocardial T_1_ values were not different between breath-hold 70° SASHA-VFA (1149 ± 27 ms), breath-hold 120° SASHA-VFA (1148 ± 22 ms), and free-breathing SASHA-HC (1156 ± 28 ms). Free-breathing 120° SASHA-VFA data processed with NAV and NORM-REG analysis had higher myocardial T_1_ values than breath-hold 120° SASHA-VFA, consistent with blood pool contamination due to inaccurate registration.

**Table 1:** Summary of T_1_ mapping parameters from 10 volunteers

	Breath-hold			Free-Breathing 120° SASHA-VFA		
	MOLLI	70° SASHA-VFA	120° SASHA-VFA	NAV	NORM-REG	HC-REG
Myocardial Mean (ms)	943.2 ± 22.2^*^	1149.4 ± 26.5	1147.7 ± 22.2	1161.7 ± 28.2^*^	1167.3 ± 27.7^*^	1156.1 ± 28.1
Myocardial Std Dev (ms)	54.7 ± 5.9^*^	72.7 ± 8.0	68.6 ± 5.9	48.0 ± 6.7^*^	45.7 ± 7.4^*^	36.2 ± 3.1^*^
Blood Mean (ms)	1503.3 ± 51.5^*^	1614.3 ± 60.9	1629.5 ± 73.8	1639.6 ± 72.9^*^	1625.6 ± 69.4	1632.6 ± 73.3
Blood Std Dev (ms)	56.0 ± 7.9^*^	97.8 ± 11.8	90.7 ± 13.8	54.8 ± 10.1^*^	43.0 ± 8.6^*^	37.3 ± 4.7^*^
Acquisition Time (s)	11.0 ± 1.2	10.0 ± 1.1	10.1 ± 1.2	107.7 ± 7.3	107.7 ± 7.3	107.7 ± 7.3
Acceptance Rate (%)	100.0 ± 0.0	100.0 ± 0.0	100.0 ± 0.0	49.3 ± 16.7	94.0 ± 13.3	94.0 ± 13.3

^*^ *p* <0.05 compared to breath-hold 70° SASHA-VFA

Standard deviations of myocardial T_1_ values were similar between 70° and 120° breath-hold SASHA-VFA (73 ± 8 ms vs 69 ± 6 ms, *p* >0.05) and higher than MOLLI (55 ± 6 ms, *p* <0.01 for both comparisons). All reconstruction techniques for free-breathing 120° SASHA-VFA T_1_ maps had higher precision than breath-hold 70° SASHA-VFA, with HC-REG having the lowest standard deviation of mean myocardial T_1_ (36 ± 3 ms, *p* <0.02). Similar trends were seen for blood T_1_ precision, but with a greater reduction in standard deviation between breath-hold 70° SASHA-VFA and SASHA-HC (91 ± 14 ms vs 37 ± 5 ms, *p* <0.01).

**Fig. 8:**
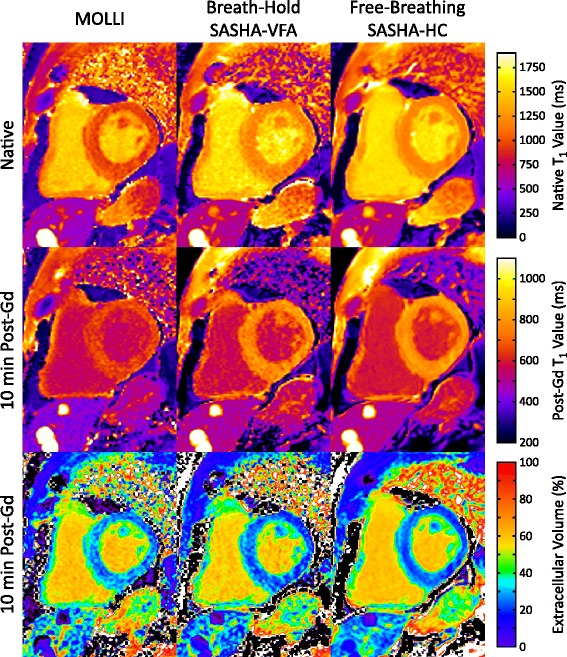
T_1_ maps from a clinical patient undergoing evaluation for coronary artery disease using MOLLI, breath-hold SASHA-VFA, and free-breathing SASHA-HC. T_1_ maps were acquired before and 10 min after administration of gadolinium contrast. ECV maps were calculated using an assumed hematocrit of 0.40

Post-contrast imaging had greater tissue-blood contrast in primary SASHA images due to shorter T_1_ values and good performance was observed for both normal and high-contrast registration techniques. T_1_ and ECV maps with MOLLI, SASHA-VFA, and SASHA-HC are shown in Fig. 8 for a clinical patient referred for evaluation of coronary artery disease. Increased precision in both native and post-contrast T_1_ maps with free-breathing SASHA-HC improves the precision of the derived ECV map.

**Fig. 9:**
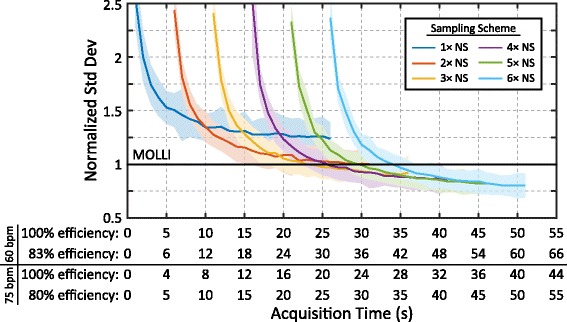
Measured myocardial T_1_ precision of free-breathing SASHA with different sampling schemes in healthy volunteers with normalization to MOLLI. The mean normalized precision (over all subjects) is shown with solid lines and the corresponding coloured shaded region indicates ±1 standard deviation. Acquisition time is shown on the x-axis for various different gating efficiencies at heart rates of 60 and 75 bpm

The relationship between myocardial SASHA-HC T_1_ precision and total imaging time measured in-vivo shown in Fig. 9 is in agreement with optimization simulations (Fig. 5c). At longer acquisition times, sampling strategies with more NS images have similar variability to strategies with fewer NS images and more TS images. At higher heart rates, the acquisition time is reduced because the number of images acquired per unit time is increased. With a heart rate of 60 bpm and an acceptance rate of 83 %, free-breathing SASHA has similar variability to MOLLI with a 30 s acquisition. With 60 s of acquisition, free-breathing SASHA had a 20 % lower standard deviation than MOLLI.

## Discussion

In this study, we developed a free-breathing SASHA-HC T_1_ mapping technique using high-contrast images to improve non-rigid image registration. The technique was evaluated in 25 volunteers and patients and SASHA T_1_ maps with high-contrast registration had higher image quality scores than T_1_ maps with navigator gated or standard image registration analysis. Free-breathing SASHA-HC, with a 108 s acquisition, had a 50 % reduction in the standard deviation of myocardial T_1_ values compared to the standard breath-hold SASHA-VFA with no bias in mean T_1_ values.

Image registration of free-breathing SASHA-HC images resulted in superior T_1_ map quality compared to both standard image registration without high-contrast images and conventional navigator gating. Accuracy of the proposed sequence was verified in phantoms and myocardial T_1_ values were not different from the previously validated SASHA-VFA sequence.

### Sampling scheme optimization

The sampling strategies investigated in this study used only non-saturated and saturation recovery images at the maximum TS time allowable by the subject’s heart rate. This allowed the greatest tissue-blood contrast, at the expense of a small reduction in precision (Figs. 4 and 5). Optimization was performed for native myocardium at 1.5 T, and further optimization should be investigated for post-contrast and 3 T imaging. Bloch equation simulations of tissue-blood contrast did not account for the inflow enhancement of blood signal. For NS images, inflow enhancement increases blood-tissue contrast for both primary and high-contrast images compared to simulations. For TS images, inflow enhancement reduces contrast in the primary images and increases contrast in the high-contrast image (Fig. 1). This is consistent with a greater than expected increase in experimentally measured tissue-blood contrast compared with simulations.

The simplicity of acquiring images with only two contrasts allows for additional flexibility in the reconstruction process. For example, within each set of NS or TS images, differences in image contrast can be assumed to only be due to motion or random noise. This information can be exploited to reject images with poor motion correction and further improve the sharpness of calculated T_1_ maps. This may be particularly useful in patients with arrhythmias or poor cardiac triggering where MOLLI-like techniques that rely on regular heart rhythms over Look-Locker sets of 3–5 images may perform poorly. Free-breathing SASHA-HC could be applied in these patients by acquiring data over a longer duration, determining the most common cardiac phase post-hoc, and discarding the remaining images.

The relationship between acquisition time and T_1_ precision is modulated by the acceptance rate, which averaged 91 % with the displacement gating approach. Lower acceptance rates are likely in patients with greater respiratory motion, although the efficiency of displacement gating was nearly double that of conventional respiratory gating of the diaphragm. As with conventional respiratory gating, acceptance rates could be further improved by coaching subjects to breath shallowly.

In-vivo data acquired with 10 NS and 50 TS images and required an average of 108 s in volunteers. However, only small improvements were found in myocardial precision with acquisition times beyond 45 s (Fig. 9). A 60 s protocol with 6 NS and 30 TS images would likely provide similar precision to T_1_ maps found in this study with allowance for lower acceptance rates. A shorter 30 s protocol with 4 NS and 10 TS images could be used in condensed clinical studies and would provide similar precision to MOLLI. These protocols assume a heart rate of 60 bpm and would be shorter in subjects with higher heart rates due to their shorter R-R duration.

The recovery duration between non-saturated images is described in heartbeats to simplify descriptions of total acquisition times. In practice, the recovery duration should be defined as a fixed number of seconds to ensure complete magnetization recovery between NS images even with high heart rates [20]. This reduces the time savings from shorter R-R intervals at faster heart rates, but avoids heart rate dependent errors in T_1_ values. At a high heart rate of 100 bpm, the 6 NS and 30 TS protocol described above would be 48 s while the 4 NS and 10 TS protocol would be 26 s.

### High-contrast image-based registration

The high-contrast image registration technique involved the acquisition of an additional 15 low-frequency k-space lines following each primary image, amounting to an additional 41 ms of imaging. The primary images are therefore acquired slightly earlier in the cardiac cycle than usual, although the temporal footprint is unchanged as the additional k-space lines are not used for the primary images. No significant artifacts due to cardiac phase motion were observed in this study, but the additional imaging time may be more problematic in patients with higher heart rates.

Tissue-blood contrast for HC images was generated using VFA modulation with high final flip angles in order to enhance the intrinsic bSSFP contrast. These high flip angles required a maximum B_1_ strength of 0.30 μT and were routinely achievable on the 1.5 T system used in this study. However, a maximum routinely achievable B_1_ of only 0.14 μT was previously reported for 3 T systems [34] and SAR limitations may further limit the maximum flip angles on higher field strength scanners. Additional optimization of the VFA modulation pattern and/or RF pulses to use lower flip angles will likely be required to apply SASHA-HC at higher field strengths. Such work may include lengthening the RF pulse duration, using variable-rate selective excitation (VERSE) [37], or using parallel transmit excitation [38] to address power and SAR limitations.

### Respiratory displacement gating

Displacement gating of respiratory motion for cardiac T_1_ mapping is an appealing alternative to traditional crossed-beam navigators of the diaphragm because the motion of the heart itself is estimated. This approach was found to be robust and had significantly higher gating efficiency than traditional respiratory navigators. As displacement gating is performed on the acquired images directly, it does not require the additional setup imaging needed for conventional respiratory navigators. Furthermore, unlike traditional navigator gating where the acceptance window is specified prior to imaging and the total imaging duration is dependent on a consistent respiratory pattern, displacement gating is performed during post-processing and the total imaging time is known in advance.

The displacement gating acceptance window was set at ±4 mm to match the ±4 mm respiratory navigator window and minimize the likelihood of errors due to mis-registration or through-plane effects. Image registration using ANTs was found to be robust when using difference images, although the acceptance window may be lowered for more challenging datasets where registration performance is worse.

Displacement gating was found to work well in short-axis image orientations where respiratory motion is primarily in-plane, allowing for very high acceptance rates. However, for slice orientations such as a 4-chamber view where respiratory motion is primarily through-plane, displacement gating may not perform well. In these cases, other metrics such as image similarity [27, 39] could be used for gating instead. These metrics can likely be applied with minimal additional modification, as the signal intensity changes between images in the NS or TS subsets are primarily driven by motion. However, as through-plane motion cannot be corrected through in-plane motion correction, a lower acceptance rate such as 50 % [27] should be used to maintain good image quality.

### Free-breathing T_1_ mapping applications

Free-breathing imaging is appealing to patients and may be more robust than breath-hold imaging in a clinical setting, as patients referred for cardiac MR examination often have difficulty performing high quality breath-holds. A 30 s free-breathing SASHA-HC acquisition with similar precision to MOLLI is an attractive alternative for clinical patients where re-acquisitions due to poor breath-holding or gating are common occurrences. Free-breathing acquisitions are also well-suited to pediatric patients, where breath-holds may be impractical or challenging.

## Conclusion

High-contrast images acquired with primary SASHA images provide consistently high tissue-blood contrast, enabling a robust image registration approach to free-breathing SASHA T_1_ mapping. Free-breathing SASHA-HC maintains excellent T_1_ accuracy and robustness to systematic confounders and T_1_ precision was higher than the reference MOLLI sequence with acquisitions longer than 30 s. Preliminary evaluation of free-breathing SASHA-HC shows promising results and warrants further study in a larger multi-center study.

## Abbreviations

bSSFP, balanced steady-state free precession; ECV, extracellular volume; GRAPPA, generalized auto-calibrating partially parallel acquisition; HC, high-contrast; HC-REG, high-contrast image registration; LV, left-ventricle; MOLLI, modified Look-Locker inversion recovery; NAV, navigator; NORM-REG, normal (without high-contrast) image registration; NS, non-saturated; SASHA, saturation recovery single-shot acquisition; TE, echo time; TR, repetition time; TS, saturation recovery time; VFA, variable flip angle

## Additional file

Additional file 1: Comparison of free-breathing SASHA-VFA images with navigator gating, normal image registration, and high-contrast image registration. (GIF 4289 kb)
