# Supplementary figures and images for: Robust free-breathing SASHA T1 mapping with high-contrast image registration
Source: J Cardiovasc Magn Reson. 2016 Aug 17;18:47. doi: 10.1186/s12968-016-0267-9 (PMC4989502; doi:10.1186/s12968-016-0267-9)

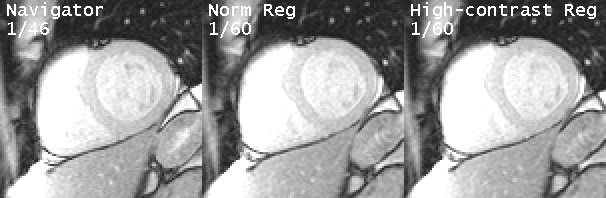

Supplement: Additional file 1: — Comparison of free-breathing SASHA-VFA images with navigator gating, normal image registration, and high-contrast image registration. (GIF 4289 kb) [file 12968_2016_267_MOESM1_ESM.gif]
